# miRNAs: Potential as Biomarkers and Therapeutic Targets for Cancer

**DOI:** 10.3390/genes14071375

**Published:** 2023-06-29

**Authors:** Atonu Chakrabortty, Daniel J. Patton, Bruce F. Smith, Payal Agarwal

**Affiliations:** 1 Scott-Ritchey Research Center, College of Veterinary Medicine, Auburn University, Auburn, AL 36849, USA; azc0142@auburn.edu (A.C.); djp0031@auburn.edu (D.J.P.); smithbf@auburn.edu (B.F.S.); 2 Department of Pathobiology, College of Veterinary Medicine, Auburn University, Auburn, AL 36849, USA

**Keywords:** miRNAs, cancer, oncomiRs, tumor-suppressor miRNAs, biomarker, therapeutics

## Abstract

MicroRNAs (miRNAs) are single-stranded, non-coding RNA molecules that regulate gene expression post-transcriptionally by binding to messenger RNAs. miRNAs are important regulators of gene expression, and their dysregulation is implicated in many human and canine diseases. Most cancers tested to date have been shown to express altered miRNA levels, which indicates their potential importance in the oncogenic process. Based on this evidence, numerous miRNAs have been suggested as potential cancer biomarkers for both diagnosis and prognosis. miRNA-based therapies have also been tested in different cancers and have provided measurable clinical benefits to patients. In addition, understanding miRNA biogenesis and regulatory mechanisms in cancer can provide important knowledge about resistance to chemotherapies, leading to more personalized cancer treatment. In this review, we comprehensively summarized the importance of miRNA in human and canine cancer research. We discussed the current state of development and potential for the miRNA as both a diagnostic marker and a therapeutic target.

## 1. Introduction

miRNAs are small non-coding RNA sequences with an average length of 18–22 bps. To date, 2654 mature miRNAs have been reported in humans [1]. miRNAs play an essential role in biological processes by regulating gene expression at the post-transcription level. miRNAs bind to messenger RNA (mRNA) in the cytoplasm, resulting in mRNA degradation or temporary inhibition of translation until needed [2]. Downregulation of a specific miRNA leads to upregulation of the corresponding proteins’ expression and vice-versa. Conversely, upregulation of miRNA leads to decreased target protein(s) expression. miRNAs bind at the 3′ and 5′ untranslated regions (UTRs) and coding regions of mRNA to induce translation repression. miRNAs are also involved in inducing gene transcription by binding within the promoter regions of a gene [3]. miRNAs are typically found inside cells; however, a portion of them are shed into circulation in lipid-coated particles known as exosomes [4]. Circulatory exosomal miRNAs have been identified as possible disease biomarkers as they are stable in blood and are protected from endogenous RNAse activity [5].

miRNAs play an important role in cancer cell transformation. miRNAs can function as tumor-suppressor genes or oncogenes by targeting genes involved in tumor development and progression or cell-cycle inhibition, respectively. Since the discovery of microRNAs, they have held great promise for cancer diagnosis, prognosis, and therapy. Different miRNA profiles can be identified for different tumor types, which could then serve as phenotypic signatures for exploitation in cancer diagnosis, prognosis, and treatment. If miRNA profiles can accurately predict malignancies, this technology could be used as a tool to overcome many diagnostic challenges [6]. In this review, we evaluated the role of miRNA in cancer diagnosis and treatment in humans and dogs separately. It is not possible to directly compare the same pathologies and miRNAs between humans and dogs. miRNA research in dogs is still at a budding stage, and there is still a lot that has not been researched. However, it is important to include the canine data because dogs are a great model for translational medicine. Furthermore, this review helps emphasize their importance as an efficient intermediate model.

## 2. Role of miRNAs in Cancer

### 2.1. Humans

#### 2.1.1. OncomiRs

**Figure 1:**
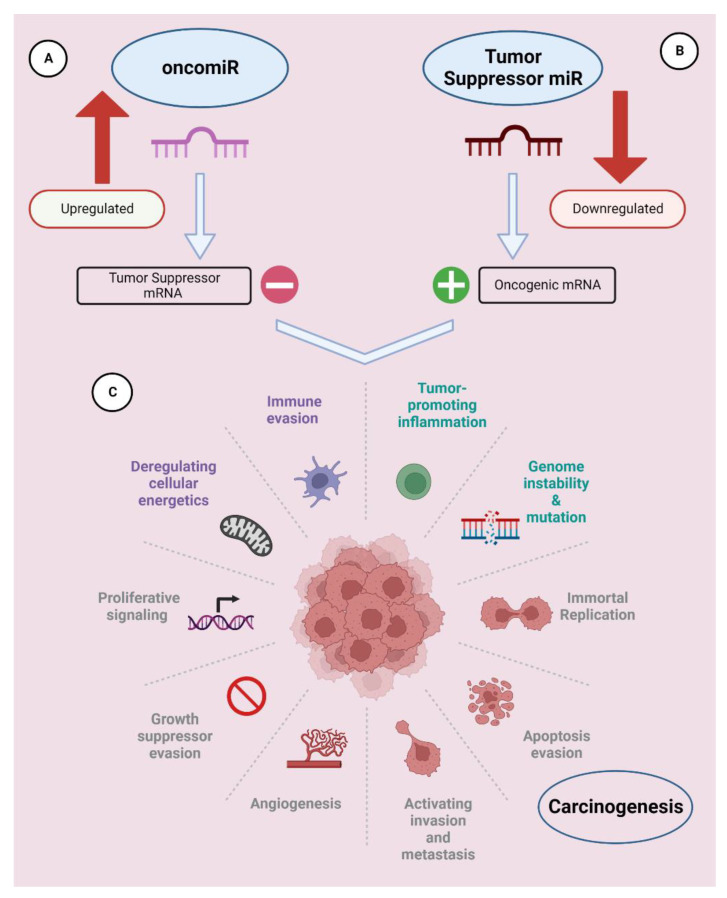
miRNAs can be classified as oncomiRs and tumor suppressors. (**A**) OncomiRs suppress tumor-suppressor gene translation and promote tumor cell growth through constitutive overexpression. (**B**) Tumor-suppressor miRNAs inhibit tumorigenesis and subsequent cancer development by suppressing the translation of mRNAs that encode for oncogenes. (**C**) Hallmarks of carcinogenesis. This figure was created using Biorender.

OncomiRs are defined as miRNAs that are overexpressed in tumors, repress tumor-suppressor mRNAs, and stimulate tumor cell proliferation and metastasis (Figure 1) [7]. There are many different oncomiRNAs with different roles in cancer growth that have been identified so far.

The miR-17-92 cluster (miRs-17, -18a, -19a, -20a, -19b, and -92a) downregulates PTEN (phosphate and tensin homolog), E2F, the transforming growth factor-β (TGF-β) signaling pathway, B cell lymphoma/leukemia 2-like protein 11 (BCL2L11), and thrombospondin-1 (TSP-1) [8]. Functionally, it favors tumor growth and is reported to be overexpressed in small-cell lung cancer, colon cancer, hepatocellular carcinoma, thyroid cancer, colorectal adenoma organoids, and renal cell carcinoma [9,10,11,12,13,14].

miR-21 is associated with phosphatase and tensin homolog (PTEN), Tropomyosin 1 (TPM1), and programmed cell death 4 (PDCD4) downregulation. miR21 overexpression is reported in a variety of cancers, such as breast, ovarian, colon, etc. [15,16,17]. Elevated levels of miR-21 were also reported in the serum, plasma, and tumor tissues in breast, lung, ovarian, colon, prostate, pancreatic, and gastric cancer patients [18,19,20,21,22,23,24,25,26,27]. Downregulation of miR-21 reduces cancer proliferation and reverses drug resistance in pancreatic, ovarian, and breast cancers [25,26,27].

miR-181 is an oncomiR that is also upregulated in various cancer types [28]. miR-181a-5p promotes breast tumor progression through N-Myc downstream-regulated gene 2 (NDRG2)-induced activation of the PTEN/AKT signaling pathway and inhibition of sprouty RTK signaling antagonist 4 (SPRY4), PH domain, leucine-rich repeat protein phosphatase 2 (PHLPP2), and inositol polyphosphate 4-phosphatase type II (INPP4B) [29,30,31]. miR-181 facilitates prostate cancer cell proliferation by targeting dosage-sensitive sex reversal, adrenal hypoplasia critical region on chromosome X, gene 1 (DAX-1) [32]. Similarly, miR-181 upregulation is associated with poor prognosis and survival in oral squamous cell carcinoma and drug resistance in melanoma [33]. miR-146a is significantly higher in plasma samples from breast cancer patients [34]. miR-221/222 is overexpressed in liver tumorigenesis and breast, colon, and pancreatic tumors [22,35,36,37].

#### 2.1.2. Tumor-Suppressor miRNAs (TS-miRNAs)

Ts-miRNSa are defined as miRNAs that downregulate cancer progression (Figure 1). The downregulation of tumor-suppressor miRNAs plays a crucial role in cancer development and proliferation [38]. TS-miRNAs are more susceptible to mutations due to their location in cancer-associated genomic regions or fragile sites. Downregulation of TS-miRNAs may occur due to dysfunctional proteins involved in their biogenesis or due to any genetic alteration [39]. Inhibition of the expression of important miRNA biogenesis machineries, such as Drosha, DiGeorge Critical Region 8 (DCGR8), and Dicer, substantially decreases miRNA production and promotes a more transformed cell phenotype [40,41,42,43,44,45].

Loss of TS-miRNA miR-16 is correlated with the progression and expansion of chronic lymphocytic leukemia, gastric, prostate, and pancreatic tumors [46,47,48,49,50]. Let-7 family miRNAs are tumor suppressors that target the Rasa and Myc oncogenes [51]. Ectopic expression of the Let-7 miRNA family induces cell death in lung cancer cells [52]. The Let-7 miRNA family is also reported to target other oncogenes, such as high-mobility group A2 (HMGA2) and MYCN [53]. The Let-7 miRNA family also acts as a tumor suppressor in breast cancer by inhibiting ERα-mediated cellular malignant growth [54].

miR-29 and miR-34 are tumor-suppressor miRNAs whose downregulated expression is associated with the progression and invasion of breast cancer, lung cancer, neuroblastoma and glioblastoma, colon cancer, stomach cancer, osteoblastoma, ovarian cancer, bladder cancer, cervical cancer, cholangiocarcinoma, melanoma, and prostate cancer [55,56,57,58,59]. miR-29 downregulation is also associated with cisplatin resistance in ovarian cancer and elevated cell proliferation in osteosarcoma [60,61,62]. Downregulation of miR-34 is associated with proliferation in pancreatic cancer, lung squamous cell carcinoma, head and neck cancer, colorectal cancer, gastric cancer, and epithelial ovarian cancer [63,64,65,66,67,68]. Elevated expression of miR-362-3p interrupts the cell cycle and inhibits tumor growth, resulting in an improved prognosis in colorectal carcinoma patients [69].

Upregulated miR-193b expression results in reduced fatty acid synthase (FASN), which in turn makes triple-negative breast cancer cells more sensitive to the effects of metformin [70]. The expression of miRNA-193b acts as a tumor suppressor in pancreatic cancer and is markedly reduced in tissues with advanced neoplasia. Cell lines transfected with miRNA-193b exhibited significantly decreased proliferation, migration, and invasiveness [71].

The impact of miRNA polymorphisms and their associated impact on cancer risk have been studied [72,73]. Single-nucleotide polymorphisms (SNPs) rs3746444 in miR-499 and rs4919510 in miR-608 are significantly associated with an increased risk of lung cancer [74]. An SNP in miRNA-499 increases the risk of prostate cancer [72]. X-inactivation-specific transcript (XIST) is a carcinogenic long coding RNA involved in ovarian tumor progression by regulating miR-355/BCL2L2 [75].

### 2.2. Dogs

Dogs have high similarity to humans in gene sequence and gene function. Dogs share the same environmental exposures and risks as humans. Almost 50% of dogs, 10 years old or older, are diagnosed with cancer at some point in their lives [76]. Due to these similarities, dogs are excellent translational models for complex human diseases, such as cancer. As in humans, miRNAs play an important role in canine cancer.

Upregulation of miRNA-19a, -19b, -17, -5p were reported in T and B cell lymphomas in dogs [77]. Additionally, miRNA-203, -181a, and -218 were reported to be underexpressed in canine lymphoma cell lines and tissues [77]. miRNA-9 enhances mast cell tumor progression [78]. miRNA-145, -203, and -205 are downregulated in canine melanoma [79,80]. miRNA-123b is significantly overexpressed in B cell chronic lymphocytic leukemia (CLL). miRNA-155 is preferentially overexpressed in T lymphocytes and some B cell CLLs, and miRNA-150 is overexpressed in T cell CLL in comparison to B cell CLL [81]. miRNA-214 promotes apoptosis in hemangiosarcoma, and miRNA dysregulation is also involved in canine splenic hemangiosarcoma [82,83]. miRNA expression profiles differ in canine splenic hemangiosarcoma, nodular hyperplasia, and normal spleens. A total of 22 miRNAs were differentially expressed in canine hemangiosarcoma samples compared to normal spleen and nodular hyperplasia [82].

In canine mammary tumors (CMTs), expression of mi-RNA-141 showed post-transcriptional downregulation of the tumor-suppressor gene family INK4A/CDKN2A [84]. miRNA-21 and -29b were reported to be upregulated in mammary gland tumor tissues, and miR-141 was reported to be overexpressed in canine mammary tumor cell lines, whereas miRNA-31, -34a, and -143/145 were reported to be downregulated in canine mammary tissues [84]. Similar miRNA expression is reported in human and dog mammary tumor patients. miR-15a and miR-16 are downregulated in canine ductal carcinomas, while miR-181b, miR-21, miR-29b, and miRlet-7f are upregulated in tubular papillary carcinomas [85]. miR-29b, miR-101, miR-143, and miR-145 expression levels were downregulated and miR-125a expression levels are upregulated in canine mammary tumors compared to normal mammary cells [86].

## 3. Role of miRNAs in Cancer Metastasis

### 3.1. Humans

Metastasis is the spread of cancer cells to a secondary site other than the primary site of tumor formation. Metastasis can either be local, through direct migration, or systemic, via the blood or lymphatics. Most deaths caused by cancer are due to cancer cell metastasis [87]. Like primary cancer, miRNAs have been noted to play a role in metastasis.

miRNA-10b overexpression is linked with the onset of breast cancer and its metastasis to the lung [88,89]. Similarly, overexpression of miRNA-320a in pancreatic cancer strongly contributes to the acquisition of characteristics, such as high levels of proliferation, invasion, metastasis, and drug resistance, as well as driving cells towards epithelial–mesenchymal transition by downregulating programmed cell death 4 (PDCD4) [90]. In glioma, miRNA-10b is highly expressed, especially in advanced-grade gliomas [91]. The inhibition of miRNA-10b produces pleiotropic effects (affects growth, invasion, and angiogenesis) in glioma cell lines but not on the growth of normal neurons and astrocytes [91].

Conversely, overexpression of miRNA-320a inhibits the capacity for invasion and migration of breast cancer in vitro, while the silencing of miRNA-320a favors it. This effect is due to the fact that miRNA-320a suppresses the powerful metadherin oncogene. Xenograft experiments (human tumor cell lines grafted in female athymic nude mice) showed that expression of miRNA-320a could inhibit breast cancer metastasis in vivo [92]. Downregulation of miR-335 causes upregulation of transforming growth factor-β (TGF-β) and its pathway members, namely Rho-associated coiled-coil containing protein 1 (ROCK1), mitogen-activated protein kinase 1 (MAPK1), and leucine-rich α-2 glycoprotein (LRG1), which leads to significant invasion and migratory potential in neuroblastoma cells [93].

### 3.2. Dogs

Similar to humans, in dogs, disease progression and metastasis are major indicators of long-term survival in many tumors. Expression analysis of 317 miRNAs from 146 canine mammary tumors revealed significantly different expression profiles between metastatic and non-metastatic groups, thus signifying the role of miRNAs as a metastasis marker [94]. The downregulation of miRNAs, including miR-29b, miR-101, miR-143, miR-145, and miR-125a, was correlated with progression to metastatic disease compared to non-metastatic canine mammary tumors [94]. miRNA-210 overexpression and miRNA-125a underexpression in canine mammary gland tumors has been shown to drive metastasis [86]. miR-124 regulates canine mammary carcinoma growth and promotes epithelial–mesenchymal transition by targeting cadherin 2 (CDH2) [95]. The hypoxia-regulated miRNA profile differs between cell lines of primary and metastatic canine oral melanoma [96]. This profiling showed that metastatic cells are more resistant to hypoxia than primary tumor cells. Downregulation of miRNAs at the 14q32 locus is associated with aggressive osteosarcoma characteristics, including increased metastatic potential and accelerated time to death in both human and canine patients [97].

Reduced expression of miRNA-34a, -134, -544, -382, -1, and -133b are linked with the invasion and migration of tumors and aggressive canine osteosarcoma [97,98,99]. miR-9 promotes a metastatic phenotype of osteosarcoma (OSA) cell lines and is upregulated in primary OSA tumors [100]. Inhibition of miR-9 reduces migration and invasion. miR-9 regulates gelsolin, which promotes enhanced motility of neoplastic cells [100]. Unlike miR-9, miR-32a reduces or inhibits canine OSA tumor and cell line growth. Metastatic OSA lesions produce 50% less miR-32a than primary tumors [99]. miRNA-433 is involved in the proliferation, invasion, and migration of dog bladder cancer cells [101].

## 4. miRNAs as Potential Cancer Biomarkers

### 4.1. Humans

**Figure 2:**
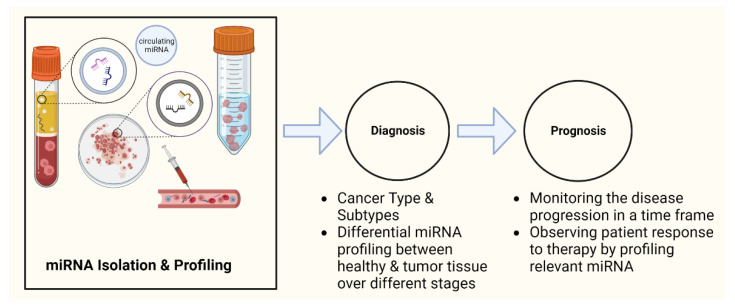
MiRNAs as cancer biomarkers: potential clinical applications. miRNAs are isolated from tumor tissue, saliva, blood, plasma, serum, etc., and then profiled for miRNA expression. miRNA profiling helps in cancer diagnosis, cancer staging, and therapeutics. This figure was created using Biorender.

The development of powerful techniques, such as microRNAome sequencing, microRNA-specific quantitative polymerase chain reaction (PCR), and in vivo antisense technologies, is expected to significantly impact clinical oncology in the near future. The characterization of sensitive and specific biomarkers, preferably those that circulate in body fluids, is critical for the timely diagnosis of cancer. Early detection of disease with a minimally invasive screening test could significantly increase the effectiveness of treatment and decrease its cost. The current understanding of the role of miRNAs in the development and progression of cancer has made miRNAs a powerful tool as a cancer biomarker. Several recent studies have revealed that miRNAs are stably detectable in plasma/serum. Due to their release into circulation and their extraordinary stability, the levels of specific miRNAs in plasma and other biological fluids can serve as diagnostic and prognostic biomarkers of diseases (Figure 2) [102].

One challenge in using miRNA as a diagnostic tool is the visualization or detection of the specific miRNA(s) to be tested. A novel DNA nanomachine was developed to selectively visualize miR-21 in cancer cells [103]. The nanomachine was designed with a zeolite imidazole framework-8 (ZIF-8) metal–organic framework (MOF), assembled with two hairpin probes (Y1 and Y2) labeled with fluorescent dye for signal amplification. After exposure to an acidic extracellular environment, the MOF decomposes and releases the hairpin molecules, which are captured by targeted miR-21. This induces catalytic hairpin assembly (CHA) amplification between the two hairpin molecules and detects the presence of the miRNA even at a low sensitivity of 27pM (64). Thus, targeting microRNAs can be an effective approach to developing sensitive diagnostics for cancer cells [104].

When the expression profiles of microRNAs were compared between healthy tissues and tumor samples, unique patterns were identified that could discern between tumor and non-tumor cells [105,106,107,108]. MicroRNA expression profiling appears to be more informative and more powerful in classifying tumor samples by their tissue of origin (something that can be complicated when tumors are diagnosed in advanced stages), tumorigenicity, and degree of differentiation than the profiles of messenger RNA (mRNA) which were traditionally used [106]. For example, a profile of only two hundred microRNAs was shown to be enough to classify poorly differentiated tumors (a frequent clinical problem) with greater precision than using the information of more than sixteen-thousand messenger RNAs [106]. Similarly, the effectiveness of miRNAs as biomarkers for tracing cancer’s tissue of origin was achieved by measuring 400 samples from 22 tumor tissues and metastases [109].

#### 4.1.1. Diagnosis

Circulating miRNA can also be an effective biomarker for cancer diagnosis. The potential of urinary miRNA biomarkers was studied for the early detection of colorectal cancer. A statistically significant increase in miR-129-1-3p and miR-566 levels in urine samples were found in primary tumor tissues compared to normal tissues [110]. MicroRNAs have been measured in additional tissues and body fluids. Plasma overexpression of miR-21, miR-125b, miR-126, miR-141, let-7, miR-205, and miR-375 were identified in prostate cancer. Similarly, overexpression of miR-20a was found in prostatic tissue, and an increase in miR-21, miR-141, and miR-375 was found in urine [111,112,113]. The identification of the overexpression of these microRNAs could be useful for the diagnosis of the disease [114,115,116].

An increase in the expression of exosomal miR-23a and miR-1246 allows the detection of disease in its early stages and differentiates between healthy and malignant tissues in colon cancer [117]. In plasma from melanoma patients, miR149-3p, miR150-5p, and miR193a-5p were reported to be higher compared to healthy patients and therefore can be used as biomarkers [118]. In saliva from esophageal cancer patients, overexpression of at least four miRNAs was reported (miR-144, miR-10b, miR-451, miR-21) versus healthy patients [119]. This fact has been corroborated in pancreatic cancer, where miR-21, miR-23, miR-181a, miR-181b, and miR-196a were overexpressed in patients’ saliva compared to controls [120].

#### 4.1.2. Prognosis

The expression profiles of microRNAs can provide important clinical information about the prognosis of patients. MicroRNA profiles correlate with survival in different tumor types, including those in early pathological states. Low levels of expression of the let-7 miRNA family and high levels of miR-155 showed a correlation with poor prognosis in lung cancer [108]. In a second study on lung cancer, high levels of miR-137, miR372, and miR-182 correlated with poor prognosis, while high levels of miR-221 and let-7a appear to be protective. In addition, the levels of this set of microRNAs were useful in predicting tumor recurrence [121]. A study on colon cancer demonstrated that high levels of miR-21 expression were associated with poor therapeutic response and poor patient prognosis [122].

A study of circulating cell-free microRNA (cf-miRNA) in ovarian cancer patients revealed that high levels of cf-miRNA (miR-92a, -200c, -320b, -320c, -335, -375, -486) are significantly associated with adverse clinical features. This suggests that a panel of cf-miRNA can work as an independent prognostic marker [123].

#### 4.1.3. Tumor Staging

It has also been suggested that microRNAs could be relevant in differentiating disease stages, including localized or metastatic cancer. Overexpression of miR-141, miR-200, and miR-375 is linked with the aggressiveness of the prostatic neoplasia [124,125]. In colon cancer, miRNA-122 and some members of the miRNA-200 family are elevated in the plasma of patients with metastatic disease with recurrence [126]. The overexpression of the exosomal miRNAs Let-7g, miR15b, miR-155, and miR-328 can potentially indicate shorter relapse in patients with advanced colon cancer stages [127]. Similarly, overexpression of exosomal miR-328 was linked with the development of liver metastasis in colon cancer patients [127]. In advanced stages, elevated levels of miR-155 indicate resistance to chemotherapy, and elevation of miR-155, miR-200c, and miR-210 indicate local recurrence, metastasis, and poor prognosis [128].

#### 4.1.4. Treatment Resistance

The usefulness of microRNAs as indicators of sensitivity or resistance to chemotherapeutic agents has been demonstrated. Overexpression of miR-34a, miR-205, and miR-31 is associated with sensitivity to taxanes. Overexpression of miR-106b has been associated with resistance to radiotherapy, while the overexpression of miR449a confers greater sensitivity to it [129,130,131,132,133,134,135]. MicroRNAs can differentiate between castration-sensitive and castration-resistant prostate cancer. In the latter, miR-125b-2 and miR-708 are decreased and miR-375, miR-141, miR-143, miR-362-5p, miR-214, let-7i and miR-545 are overexpressed [131,135].

Downregulation of miR-452 is linked with acquired resistance to the drug Adriamycin (ADR) or doxorubicin. Upregulation of miR-452 decreased resistance to ADR in MCF7 cells by abrogating the activity of IGF-1R [136].

It has also been shown that the loss of miRNA-200c correlates with a better prognosis in breast cancer and leads to increased cancer cell sensitivity to doxorubicin by reducing multidrug resistance mutation (MDR1) gene expression [137]. Recent studies have suggested that negative regulation of miR-542-3p may contribute to resistance to the breast cancer drug trastuzumab (Herceptin) since the drug induces the expression of this miRNA in SKBR3 and MCF7/Her2 cells by activating the PI3K-AKT pathway and generating a blockade of the G1/S checkpoint [89]. miRNA-205, at high levels, is predictive of a better response to the TAC NAC regimen (docetaxel, doxorubicin, plus cyclophosphamide). This miRNA improves the chemosensitivity of cancer cells to TAC by suppressing both vascular endothelial growth factor A (VEGFA) and fibroblast growth factor 2 (FGF2). It is downregulated in MCF-7/A02 and CALDOX cells (two drug-resistant derivatives of MCF-7 and Cal51 cells). Thus, the increase in miR-205 can serve as a predictive biomarker and a potential therapeutic target for the treatment [138].

miR-21 is involved in the development of resistance to gemcitabine through epithelial-to-mesenchymal transition (EMT) and AKT pathway activation. PTEN, a direct target gene of this miRNA, is significantly downregulated in drug-resistant breast cancer cells, and restoration of PTEN expression blocks miR-21-induced EMT and gemcitabine resistance [139].

Aggressive triple-negative breast cancers (TNBC) rapidly develop resistance to chemotherapies. Genotoxic treatments, such as doxorubicin (Dox), were found to significantly increase the expression of miR-181a in TNBC cells, which increases metastasis. Regulation of this miRNA is orchestrated by the transcription factor STAT3. BCL2-associated X (BAX) was also identified as a direct functional target of miR-181a. BAX suppression decreases apoptosis and promotes cell invasion, which is critical in promoting therapeutic resistance and aggressive behavior of TNBC cells after genotoxic treatment. An antagonist of this miRNA may serve as a promising strategy to sensitize TNBC cells to chemotherapy and mitigate metastasis [140].

miR-182, found in triple-negative breast cancer tissues, induces proliferation and invasion of MDA-MB-231 cells through negative regulation of the PFN1 protein. Treatment strategies using miR-182 expression inhibition or PFN1 gene overexpression could also benefit patients with TNBC [141]. High levels of miR-621 in breast cancer predict a better response to chemotherapy since the ectopic overexpression of this miRNA promotes apoptosis and chemosensitivity to drugs by suppressing FBXO11, which causes an increase in p53 activity, promoting apoptosis in cells exposed to chemotherapeutic agents [142].

The role of miRNA-340 was examined using a lentiviral vector overexpressing miRNA-340 in the triple-negative breast cancer cell line MDA-MB-231. Overexpression of miR-340 decreased the expression of the transcription factor SOX2 and cyclin-dependent P16 and P27 kinase inhibitors. [143]. miR-340 inhibition of these breast-cancer-associated genes has potential therapeutic use.

An analysis of almost 600 miRNAs in tumors from patients with progressive bladder cancer was performed. The patients were treated with cisplatin, and it was found that 15 miRNAs play an important role in the response, 5 in the survival interval, and 3 in both aspects [144].

### 4.2. Dogs

Circulatory exosomal miRNAs are being studied for their utility as markers for early detection in cancer screening tests using a minimally invasive liquid biopsy approach in dogs [103]. miRNA expression differs between tumor type and stage, which allows for more accurate diagnosis of a wide variety of canine tumors [86]. A recent study using next-generation sequencing and PCR amplification looking at thirteen canine cancer cell lines identified six miRNAs elevated in all cancer types. Of these six miRNAs, cfa-miR-9 was the most overexpressed in all cell lines compared to normal cells [103]. Another study showed that miR-216 and miR-126 showed different circulatory levels depending on tumor type. miR-216 showed elevated levels in the blood of sarcoma patients, while miR-126 showed elevation in epithelial-derived tumor patients [145]. The identification of tumor-specific miRNAs could be an important diagnostic tool in splenic masses where ~75% are malignant, with concurrent poor prognosis (median survival is 110 days) versus the remaining 25% where median survival is over one year [146].

A number of miRNAs have been identified as specific biomarkers in tumor tissues in several tumor types in dogs. For example, miR-103b, -34a, -106b, and miR-16 can potentially be used as diagnostic biomarkers for transitional cell carcinoma of the bladder in dogs [147,148]. Circulatory miR-214 and -126 are reported as strong predictors of disease-free intervals and overall survival in appendicular OSA patients receiving amputation and chemotherapy [145]. Circulatory miRNA-214 and -126 are also reported as potential biomarkers for the diagnosis and prognosis of osteosarcoma and other canine neoplastic diseases [145].

Cutaneous mast cell tumor is a common neoplasm in dogs. Dysregulation of 63 miRNAs (18 upregulated and 45 downregulated) was reported in mast cell tumor tissues compared to adjacent healthy tissues. miR-21, miR-379, and miR-885 are promising biomarkers to identify patients with mast cell tumors and lymph node metastasis [149]. Canine mammary carcinoma patients have differentially expressed miRNAs. Circulatory miR-19b and miR-18a can potentially be used as biomarkers for diagnosis and prognosis in canine mammary cancer patients [150]. Similarly, miR-21, miR-155, miR-9, miR-34a, miR-143/145, and miR-31 have altered (upregulated and downregulated) expression in canine mammary tumor cells and may serve as potential targets for breast cancer therapeutics [84].

The development of accurate blood tests based on miRNA expression could provide veterinarians with more information for more effective treatment. Minimally invasive liquid biopsy in canine multicentric lymphoma is proposed to evaluate the potential outcome of chemotherapy. Circulatory miR-205, miR-222, miR-20a, and miR-93 can be used as molecular signatures to predict non-responsive cases [151]. Similarly, circulatory miRNAs can also be used as biomarkers to depict cardiotoxicity in dogs due to the prolonged use of the chemotherapeutic drug doxorubicin. miR-502 downregulation is a potential biomarker to predict cardiotoxicity before other echocardiographic parameters [152]. Exosomal miR-143 and miR-221 are significantly increased in metastatic melanoma patients and can be used as biomarkers to identify metastatic melanoma [153]. miR-15b and miR-342-3p isolated from plasma can be used as non-invasive biomarkers to differentiate glioma from other intracranial diseases in dogs [154]. miR-214 and miR-126 are oversecreted in malignant endothelial proliferated diseases [83]. Therefore, these miRNAs have potential as diagnostic biomarkers for malignant endothelial proliferated diseases in dogs.

Uveal melanoma is a primary intraocular tumor in dogs with no effective means of predicting metastasis. Fourteen miRNAs have been identified that exhibit significant differences between metastatic and non-metastasizing tumors. miRNA expression profile of these 14 miRNAs may be used to predict metastasis and for therapeutic purposes [155].

Research on canine tumors has implications in human medicine; however, canine miRNAs are much less well studied than human miRNAs. There is a significant crossover in miRNAs identified in the two species [156]. In mammary tumors, a study showed nine of ten overexpressed miRNAs in canine tumors are the same as in human mammary tumors [85]. Circulating miR-214 and miR-126 showed similar patterns in human and canine patients based on tumor type and disease progression [145]. Downregulation of miR-1 and miR-34 and upregulation of miR-9 and miR-106b clusters have been correlated with increased proliferation and invasion of osteosarcoma in humans and dogs [156]. The crossover between canine and human miRNA expression patterns in tumors poses a unique research opportunity. The similarities allow for easy interspecies application and the advancement of diagnostic technology and treatments in both species simultaneously.

## 5. Role of miRNAs in Cancer Treatment

### 5.1. Humans

As stated above, each cancer possesses a specific combination of miRNAs, either overexpressed oncomiRNAs targeting tumor-suppressor genes or downregulated tumor-suppressor miRNAs targeting oncogenes [157]. This profile of expressed miRNAs may be used to establish a “fingerprint” that could potentially identify specific tumor types and even subtypes with a given tumor. Since miRNAs are involved in cancer cell gene regulation, these may provide excellent opportunities to design personalized therapeutics for cancer patients. miRNA-based anti-cancer therapies have recently generated interest either as monotherapies or in combination with other cancer therapies. Targeting oncomiRNAs induces the expression of tumor-suppressor genes, which in turn enhance tumor cell killing and promote tumor regression [158]. However, physiological and cellular barriers hamper the in vivo efficacy of anti-miRNA technologies.

As previously mentioned, one of the first miRNAs detected in the human genome, miR-21, is overexpressed in glioblastoma [159] and could be used as a therapeutic target in this type of cancer. In glioblastoma cells, the additive interaction of antisense oligonucleotide inhibitors to both miRNA-21 and miRNA-10b may constitute an effective therapeutic strategy to control glioblastoma growth by inhibiting oncogene expression and inducing tumor-suppressor gene expression. miRNA-21 inhibitors also interrupt the activity of the EGFR pathway, thereby increasing the expression of PDCD4 and Tropomyosin 1 (TPM1) and reducing the activities of matrix metalloproteinases (MMPs) [159]. Inhibition of NADPH oxidase (NOX) dramatically lowered the invasive potential of lung cancer in vitro by decreasing miRNA-21-expression [160].

When miRNA inhibitors are co-administered with an anti-cancer agent, they can induce synergistic effects (e.g., in glioblastoma) [161]. A concern in miRNA modulation strategies is the proper identification, in silico, of miRNA inhibitors or analogs that can effectively inhibit or mimic the function of specific miRNAs to achieve miRNA loss or gain of function, respectively. Another challenge to miRNA-directed therapies’ efficiency is the long-term release of these miRNA inhibitors or mimics at their specific target sites. A new form of miRNA inhibitor delivery has been developed to answer these concerns, specifically targeting miRNA-155 in an acidic tumor microenvironment in murine lymphoma. To achieve this, peptide nucleic acid anti-miRs were attached to a peptide with a low pH-induced transmembrane structure (pHLIP). This construct could target the tumor microenvironment and transport anti-miRs under acidic conditions across the plasma membrane. This approach evades the hepatic barrier (removal of foreign proteins from circulation by the hepatic reticuloendothelial system) and facilitates miRNA targeting through a non-specific endocytic pathway [162]. An alternative miRNA inhibitor delivery strategy using R3V6 peptide was evaluated as a transporter of antisense oligodeoxynucleotides [163]. Serum stability assays showed that R3V6 protected miRNA inhibitors from nucleases more efficiently than polyethyleneimine (PEI; 25 kDa, PEI25k). In an in vitro transfection assay, R3V6 transported antisense oligodeoxynucleotide anti-miRNA-21 into cells more efficiently than PEI (25 kDa, PEI25k) and lipofectamine [163].

microRNA can also serve as a candidate for developing oncolytic virotherapy. A new miRNA-modified Coxsackievirus B3 (CVB3) was developed by inserting miR-145/143, miR-1, and miR-216 target sequences into the 5′ untranslated region (5′ UTR) of the CVB3 genome. miR-145/143 is downregulated in tumors, miR-1is muscle-specific, and miR-216 is pancreas-selective [164]. The virus is downregulated in any cell expressing any of the three miRNAs but is replication-competent in cells, such as a tumor, that do not express any of the three. This novel miRNA-modified oncolytic virus inhibited triple-negative breast cancer growth in immunocompromised mouse models [164].

Chemotherapy and miRNA therapy combinations have shown synergistically increased antineoplastic activities. The combination of a miRNA-21 inhibitor and Taxol is an effective therapeutic strategy to control the growth of glioblastoma multiforme (GBM) by inhibiting the expression and phosphorylation of STAT3 in vitro [165].

### 5.2. Dogs

Canine hemangiosarcoma has an extremely poor prognosis. Upregulation of miR-214 induces apoptosis in hemangiosarcoma cell lines. Intraperitoneal administration of synthetic miR-214 (miR-214/5AE) exhibits anti-tumor effects in a murine model of canine hemangiosarcoma. It induces apoptosis and prohibits cell proliferation [166]. Similarly, intratumoral administration of synthetic miRNA-205 (miR-205BP/S3) can be used to treat canine malignant melanoma. Administration of miR-205BP/S3 in eleven dogs led to five complete remissions, three dogs with stable diseases, and three cases of progressive disease [167].

Novel miRNA vectors are being explored to induce oncolysis and disease remission in solid tumors leading to a new wave of cancer treatments [168]. Canine osteosarcoma patients with metastatic disease have poor prognosis. miR-34a suppresses the oncogene Eag-1, and the downregulation of miR-34a has been correlated with the progression of canine osteosarcoma. In vitro and in vivo models showed that administration of miR-34a inhibited osteosarcoma progression and decreased Eag-1 production [101]. A bioengineered miRNA prodrug (tRNA/miR-34a) was successfully processed into mature miR-34a in canine osteosarcoma cells. The administration of tRNA/miR-34a in murine models with canine OSA xenografts caused delayed tumor growth, increased necrosis and apoptosis, and reduced cellular proliferation [168]. tRNA/miR-34a treatment showed 32% less tumor growth and more prolonged survival versus the control groups [168]. The emergence of new research on the effects of miRNA on tumor progression allows for novel treatment development.

## 6. Future Perspective and Summary

As stated above, a wide range of miRNAs can be utilized as cancer biomarkers and for cancer therapeutics. A cancer diagnosis is often delayed to the point that cancer has progressed significantly and metastasis has occurred. miRNAs show promise as a biomarker for earlier cancer detection and, therefore, more effective treatment. miRNAs can be secreted into the bloodstream, are stable as circulating free miRNAs, or are protected from endogenous RNAse enzyme activity by encapsulation in exosomes or proteins. These biomarkers can serve to screen patients for potential undiagnosed tumors. A positive result would trigger a search for the tumor and potential therapy to treat the primary tumor and reduce the chance of metastases. miRNAs may potentially serve as markers for staging tumors, including the presence or absence of metastases, based on the stage of disease at the time of sample collection. All of this is achievable using minimally invasive, “liquid biopsy” approaches to monitor tumor progression, response to therapy, and relapse. miRNAs are involved in cancer progression and metastasis in both humans and dogs. Given the very high degree of sequence similarity between human and dog miRNAs, the miRNAs identified in dogs will almost certainly have similar or identical functions and properties in people. This will allow the miRNAs researched and explored in dogs to be rapidly translated into humans. However, to realize the full potential of miRNAs in these areas, more research is required regarding the use of miRNAs as therapeutic biomarkers in dogs and humans.

## Data Availability

Not Applicable.
